# A response to Yu et al. "A forward-backward fragment assembling algorithm for the identification of genomic amplification and deletion breakpoints using high-density single nucleotide polymorphism (SNP) array", BMC Bioinformatics 2007, 8: 145

**DOI:** 10.1186/1471-2105-8-394

**Published:** 2007-10-16

**Authors:** Oscar M Rueda, Ramon Diaz-Uriarte

**Affiliations:** 1 Statistical Computing Team, Structural Biology and Biocomputing Programme, Spanish National Cancer Center (CNIO), Melchor, Fern'andez Almagro 3, Madrid, 28029, Spain

## Abstract

**Background:**

Yu et al. (BMC Bioinformatics 2007,8: 145+) have recently compared the performance of several methods for the detection of genomic amplification and deletion breakpoints using data from high-density single nucleotide polymorphism arrays. One of the methods compared is our non-homogenous Hidden Markov Model approach. Our approach uses Markov Chain Monte Carlo for inference, but Yu et al. ran the sampler for a severely insufficient number of iterations for a Markov Chain Monte Carlo-based method. Moreover, they did not use the appropriate reference level for the non-altered state.

**Methods:**

We rerun the analysis in Yu et al. using appropriate settings for both the Markov Chain Monte Carlo iterations and the reference level. Additionally, to show how easy it is to obtain answers to additional specific questions, we have added a new analysis targeted specifically to the detection of breakpoints.

**Results:**

The reanalysis shows that the performance of our method is comparable to that of the other methods analyzed. In addition, we can provide probabilities of a given spot being a breakpoint, something unique among the methods examined.

**Conclusion:**

Markov Chain Monte Carlo methods require using a sufficient number of iterations before they can be assumed to yield samples from the distribution of interest. Running our method with too small a number of iterations cannot be representative of its performance. Moreover, our analysis shows how our original approach can be easily adapted to answer specific additional questions (e.g., identify edges).

## Background

The recent paper by Yu et al. [1] proposes a new method for the analysis (segmentation) of high density single nucleotide polymorphism (SNP) arrays to detect copy number changes (CNAs) in genomic DNA. Their approach has been designed to be highly sensitive for edge detection and is tailored to SNP arrays. In their paper, Yu et al. compare the performance of their approach with that of several alternative methods initially developed for the analysis of array-CGH (aCGH) data. The methods compared include Circular Binary Segmentation [2], GLAD [3], CGHseg [4] – though not in the original formulation of their authors, as the recommended adaptive penalization of [4] is not used–, and three Hidden Markov Model approaches: a homogenous one [5], the non-homogeneous one of Marioni et al. [6], and RJaCGH, our own non-homogeneous HMM using Markov Chain Monte Carlo (MCMC) with Reversible Jump [7].

The different aCGH technologies, from BAC-based aCGH to oligonucleotide aCGH (oaCGH), including Affymetrix SNP arrays, differ in several ways, both in terms of coverage, technology, noise per individual element, requirement for reference samples and minimal DNA quality, and LOH information returned (e.g., see [8-13]). Some of these differences might require customized approaches for SNP-based arrays [11,13].

Thus, our objective here is not to show the optimality of RJaCGH for SNP-based arrays but, rather, to reanalyze the data of Yu et al. [1] using more appropriate parameters for the Markov Chain Monte Carlo in RJaCGH, which yield much improved performance, as well as to show extensions of our model for edge detection, which show the relevance of model-based methods of direct interpretation that explicitly return probabilities.

### Re-analyzing the data

We will re-analyze the data in [1]: in the original comparison RJaCGH was used with a) the incorrect reference for normal samples and b) too few iterations for both the burn-in and posterior sampler in the MCMC algorithm.

The data analyzed, in the non-altered condition, have a mean value of 1.03 and a median of 1.05. RJaCGH, by default, expects that the "normal" values will be centered around 0. This value of 0 is, often, the result of the log2 of a (normalized) ratio of 1. Expecting values of non-altered regions to be centered around 0 is not unique to RJaCGH; for instance, Fridlyand et al. [5], in p. 138, explain why they expect that the median copy number of the array will typically be normalized to 0. Likewise, Marioni et al. [6] assume a Gaussian for the log2 ratios of each state, which therefore means that regions with unaltered copy number are expected to show values centered around 0. Similarly, Picard et al. [4] indicate (p. 2) "Array CGH data are normalized with a median set to log2(ratio) = 0 for regions of no change (...)". Now, the last three methods, CGHseg [4], aCGH [5], and BioHMM [6], might not be adversely affected, in terms of edge detection, if the normal samples, instead of being centered around 0, are centered around 1 (as in the current data). However, RJaCGH is adversely affected: our algorithm, by default, uses a step where clones are separated into the three groups "gained", "lost", "no change", and information about the expected value of the non-changed clones is used to separate these three states, and therefore to identify some of the edges. Appropriately using RJaCGH, thus, requires either normalizing the data so that unaltered areas are centered around 0 or, else, explicitly using the "normal.reference" parameter to the RJaCGH function in our code (this parameter might not have been available in the version used in [1], but recentering the data would have had the same effect).

Second, and more importantly, RJaCGH has been run with a burn-in run of 50 iterations and results obtained from 450 runs (TOT parameter set to 500). Except for extremely simple Bayesian models, in real-life usage, much longer runs are often necessary (e.g., [14]) both for the burn-in period (i.e., to try to ensure that we are sampling from the true posterior) and for the number of MCMC iterations used after the burn-in period (i.e., the number of samples used to actually carry out inferences). The number of iterations should be even larger when we are using Reversible Jump and a complex model as HMM [15-17]. It is extremely unlikely that with only 50 burn-in iterations our model would be anywhere near convergence, and thus all inferences from these models are suspect.

### Parameters for re-analyzing the data

The data and further details on the analysis and simulations were kindly provided by the first author of the paper, Tianwei Yu. We rerun the RJaCGH analysis, using 10000 burn-in iterations and 40000 samples for the posterior. Note that, to depart from the original [1] as little as possible, we do not use multiple chains in parallel (which would, however, be the recommended procedure and would also allow to check for convergence [14,18], as explained in our paper [7]).

As explained above, we set the reference mean to 1 (parameter "normal.reference" in RJaCGH). We could have, instead, centered all data to ensure that the non-altered regions were centered around 0. To account for different levels of noise in the data (or to modify the trade-off between false positive and false negative rates) RJaCGH uses only one parameter, "normal.ref.percentile". This parameter might not have been easily available to the authors of [1] and, thus, none of our key criticisms hinge around non-usage of this parameter. "normal.ref.percentile" is used to set the width of the confidence band based on a Normal (Gaussian) distribution: higher values of "normal.ref.percentile" will tend to incorporate more states into the "non-change" final state. We have varied "normal.ref.percentile" from 0.5 to 0.99 to construct ROC curves as in Figure 3 of [1]. All code for our analysis is available from [19].

## Results

**Figure 1:**
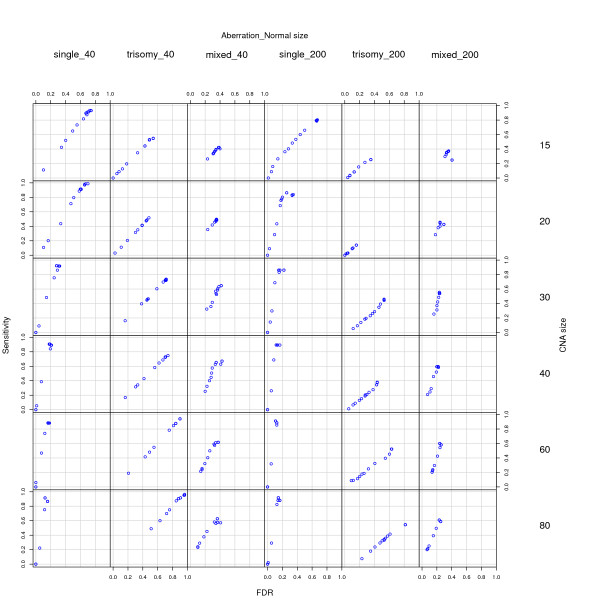
Reanalysis of simulations using RJaCGH. This figure is equivalent to Figure 3 in [1], but with RJaCGH run for a suitable number of iterations and with appropriate parameter for the reference value. As in [1], every point plotted is the mean of 100 simulated chromosomes and each chromosome had six normal and five altered segments. Ten values of "normal.ref.percentile", from 0.5 to 0.99 in steps of 0.05, were used to construct the ROC curves. See text for details.

Figure 1 shows our re-analysis using RJaCGH. As can be seen from the figure, in virtually all cases the results with appropriate parameters are much better than those shown in Figure 3 of [1] and the results are clearly competitive with those of other methods. For single aberrations and normal size 200–except with CNA size 15– and trisomies and normal size 40 and (second and fourth columns of the figure) RJaCGH is the best performing method as it is the method with values closest to the upper left corner of the figures. Its performance is comparable to that of the best performing methods with a single aberration and normal size 40 (first column). RJaCGH is a bad performer with the mixed scenarios (columns three and six). Finally, the results from trisomies and normal size of 200 are difficult to compare among methods as this is a scenario where all methods perform poorly: overall, RJaCGH is the method that returns values which are closer to the upper left corner of the figures, but the overall Sensitivities are never large whereas some other methods can achieve higher Sensitivities but at the cost of very large False Discovery Rates. In summary, when using appropriate parameters (number of iterations of the sampler and burn-in, as well as the correct reference for normal values) RJaCGH is a competitive method for the analysis of these data.

### Searching directly for edges

In the reanalysis above, we used RJaCGH in the default way. This means that the inference about the edges is not one where we estimate the probability of a spot being an edge. As RJaCGH is an HMM-based model, its main objective is to try to assign clones to their true (hidden) state; RJaCGH additionally automatically collapses the hidden states to three biologically-motivated levels: gained/lost/no-change. Thus, to find edges, we first use RJaCGH to infer the status of a clone (gained, lost, no change) and then we identify the edges by looking for two consecutive probes where status changes. There is, however, a simple and direct procedure to obtain the probability of an edge that does not involve the above indirect procedure. Since we can apply the Viterbi algorithm to every MCMC sample [7], for each MCMC sample we can directly obtain the locations where there is an edge: spot i is an edge if its state –as identified by the path from the Viterbi algorithm– is different from that of spot i + *1*. This approach has the advantage of returning the probability of an edge in a given spot, directly incorporating averaging over number of hidden states, and not requiring to specify "normal.ref.percentile". The later also illustrates one difference with regards to the previous approach. In the previous approach, the edges are identified from the "collapsed" classification of all hidden states into gained/lost/no-change (and this is why we required the specification of "normal.ref.percentile"). Here, in contrast, we will identify as an edge any change in state, even if the change in state is between hidden states that will eventually be considered a single biological level in the classification (e.g., if the change in state is between two different hidden states that will later be both considered "gain").

**Figure 2:**
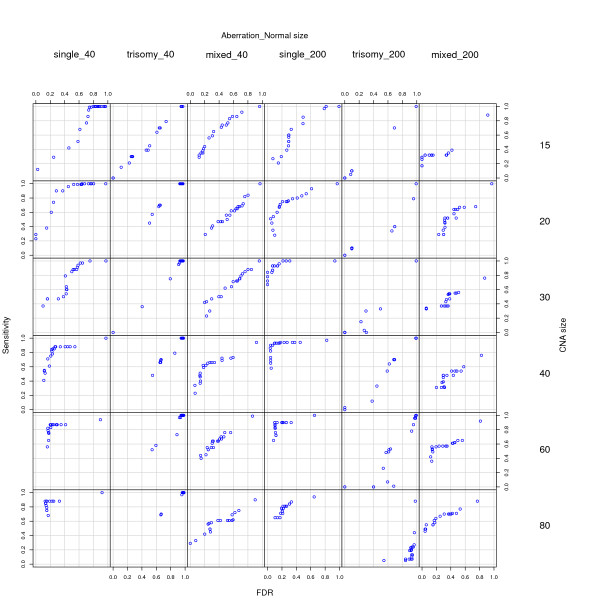
Reanalysis of simulations using RJaCGH with edges from Viterbi. This figure is equivalent to Figure 3 in [1], but with RJaCGH run for a suitable number of iterations and with appropriate parameter for the reference value. As in [1], every point plotted is the mean of 100 simulated chromosomes and each chromosome had six normal and five altered segments. In contrast to Figure 1, the location of edges is obtained directly from the Viterbi run in each MCMC sample. See text for details.

**Figure 3:**
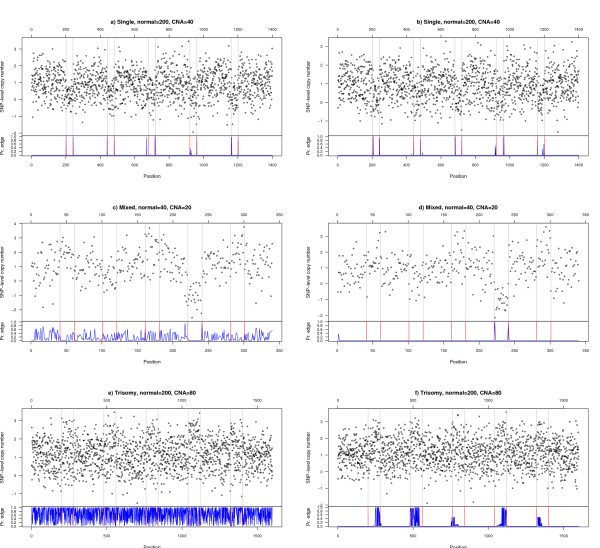
Probability of edge vs. true edges. Six sample cases where we show the probability of there being an edge in each spot (from the Viterbi algorith –see text) in blue, together with the observed data and the location of the true edges (red and gray lines).

We run the analysis again, to replicate Figure 3 in the original [1]. As with this procedure we obtain the probability of an edge, for ROC curve construction we will declare that a spot is an edge if the probability is above a given threshold, and will generate the ROC curves by changing this threshold. We have varied the threshold from 0 to 0.95 in steps of 0.05 (so the ROC curves are based on 20 threshold values). The results are shown in Figure 2. As can be seen, the results are in most cases slightly better than those in Figure 1 and, thus, also sustain the conclusion that RJaCGH is a competitive method for the analysis of these data. To further illustrate this procedure, and to understand some of the results shown in Figure 2, Figure 3 shows six case examples. Panels a) and b) correspond to a situation (single alteration, normal size of 200 and CNA size of 40) which is among the best performing. In both cases, the probability of an edge is maximal exactly at, or very near to, the spot where the actual edge is located, and there is a very clear separation between spots where there is some probability of an edge and the rest of the spots. Panels c) and d) identify an intermediate case (mixed aberration, normal size of 40, CNA size of 20). In panel d), only two true edges are clearly detected (corresponding to a low sensitivity case), whereas in panel c) in addition to the two true edges with high probability, there are many spots with intermediate probability, which leads to an increase in Sensitivity with FDR almost along the diagonal line as we lower the threshold (since most of the spots with intermediate probabilities of being edges are not true edges). Panels e) and f) are example of one of the worst performing scenarios (trisomy, normal size of 40 and CNA size of 20); panel e) is a usual example where there are many changes in state, very few of which occur at the true location and we, thus, have very low Sensitivity and high FDR; in panel f) we still see many changes in state, but many of the true locations can be identified if we are willing to pay the price of a high FDR. Interestingly, and since the algorithm for RJaCGH incorporates the Viterbi algorithm as part of its computations, finding edges in this way only requires a minimal amount of coding (about 35 lines of C code and 30 lines of wrapper R code).

Finally, there is no need to re-run the algorithm to modify the trade-off between sensitivity and FDR, since this trade-off can be changed by changing the probability threshold over which we consider a spot to be an edge. This feature provides added flexibility, which allows users to choose the best threshold for any application, or even to use different thresholds for different probes or chromosomes. Moreover, even if the edge is not located precisely, we can obtain evidence suggestive of the area where an edge is located. For instance, in Figure 3, panel f) the exact location of any edge is not well defined, but there are three areas where spots of high edge probability are located, and an additional two with areas of medium edge probability; these types of representations might suggest refining searches in these areas. In contrast, panel e) is indicative of a situation where the algorithm is likely to be performing poorly, thus alerting users that those results should not be trusted.

## Discussion

We have shown (Figure 1) that, when used properly (i.e., with realistic number of chains for the MCMC algorithm and with the correct reference for the normal–no-change–probes), RJaCGH can achieve performance on-par with, or better than, other methods in this data set. Moreover, and in contrast to other methods, the model of RJaCGH is sufficiently flexible that we can directly obtain answers to the questions we are interested in, as shown in Figure 2, where we directly obtain the probabilities of an edge from the algorithm, without intermediate and indirect calculations. In fact, one of the advantages of the explicit model underlying RJaCGH [7] is the possibility of obtaining answers (in the form of statistics, posterior probabilities, etc) to the biologically relevant questions. Our original code was targeted to correctly classifying probes into the gained/lost/no-change status but we can, as well, focus the analysis on the probability that a probe is an edge. Moreover, it is easy to extend this approach to ask (and answer) more specific questions; for example, we could be interested in the probability that a probe is an edge between a state of no-change and a state of gain. In contrast, answers to these types of questions are often hard to obtain from other methods and the answers, if available, are rarely as immediately applicable as a probability. Finally, both our method and BioHMM [6] are unique among the methods compared because they can incorporate unequal distance between probes. With the data provided, the performance advantage of these two methods can not be detected since, by construction of the simulations, the inter-probe distance is the same (or plays no role in the probability of change in state).

A broad issue raised by the paper of [1] and our reanalysis is why HMM-based models, as well as approaches such as CGHseg and Circular Binary Segmentation, do not achieve better performance with these data, especially given their excellent performance in other aCGH platforms [7,20,21]. The comparatively poor performance of these approaches with SNP-based data might be attributable more to specifics of the Affymetrix platform [11-13,22-24] than to specific values of parameters related to noise in the data (as exemplified by Figure 3).

## Abbreviations

aCGH, array-based comparative genomic hybridization; CBS, aCGH analysis method developed by [2];CGHseg, aCGH analysis method developed by [4]; GLAD, aCGH analysis method developed by [3];MCMC, Markov Chain Monte Carlo; oaCGH, Oligonucleotide aCGH; RJaCGH, Reversible Jump-based analysis of aCGH data developed by [7]; ROC (curve), Receiver operating characteristic (curve); SNP, Single nucleotide polymorphism;

## Authors' contributions

OMR implemented most of the original method. RDU drafted the MS and carried out most comparisons. Both authors modified and approved the ms.

## Response from original authors

Tianwei Yu & Xiaofeng Zhou

Address:

Department of Biostatistics, Rollins School of Public Health, Emory University, Atlanta, GA USACenter for Molecular Biology of Oral Diseases, College of Dentistry, University of Illinois at Chicago, Chicago, IL USAGuanghua School & Research Institute of Stomatology, Sun Yat-Sen University, Guangzhou, China

Corresponding author: Xiaofeng Zhou

xfzhou@uic.edu

In our paper [1], the comparison study was done with a previous version of RJaCGH (version 1.0.0). The current version of RJaCGH (version 1.1.1) was publicly available only after our paper was already published. Two key parameters were non-existent in the version 1.0.0 [25]. They are "normal.ref.percentile" and "normal.reference". The first one controls the sensitivity in edge detection, and the second one determines the expected value of the non-changed clones.

As Rueda and Diaz-Uriarte pointed out, two factors contributed to the apparent suboptimal performance of RJaCGH in our original paper [1]. The first is the expected value of the two copy clones being non-zero. It is a characteristic of copy number data processed by Copy Number Analysis Tool (CNAT) from Affymetrix Inc. In the version of RJaCGH we tested, there was no such parameter to adjust and its relevance would not have been known to the user. Obviously the RJaCGH package has been improved afterwards to add the parameter "normal.reference" to address this issue. The other two HMM-based packages already reached excellent performance without setting such a parameter [1]. We wish to point out the difficulty and importance of correctly setting this parameter in Affymetrix SNP array-based copy number analysis. With the current Affymetrix SNP array platforms, the normal reference dataset was provided by an internal library, which is implemented as an integrated part in Affymetrix CNAT software [22]. While this approach greatly reduces the effort and cost to perform copy number analysis, it could also introduce bias from various sources. Hence even minimum variations in the experimental procedure may cause the median value of the two copy clones to drift from the ideal value. In addition, the X chromosome without copy number deviation from a male sample will yield copy numbers centered around 1, as opposed to 2 from the autosomes. These issues require special care by the user when a "normal.reference" value needs to be set.

**Figure 4:**
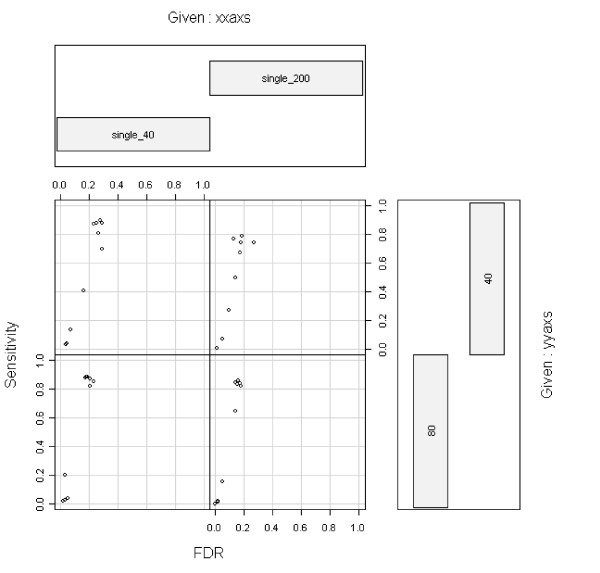
Sensitivity and FDR using burnin = 50, TOT = 500, and k.max = 3.

The second factor contributing to the suboptimal performance was the substantially reduced number of iterations. Due to the high computational cost of the method (Table 2 of our paper) [1], we conducted simulations using reduced number of iterations based on our empirical observations on RJaCGH 1.0.0. As we listed in table 1 of our paper [1], the number of states was set to 3 when all five CNA segments are of the same copy number. This is different from what Rueda and Diaz-Uriarte used in their current paper. It results in a relatively simple chain. Thus the impact of reduced iterations would be small for these cases. With the new version RJaCGH 1.1.1, we picked four scenarios where the package was successful and tested the effect of the reduced number of iterations using the code by Rueda and Diaz-Uriarte. The performance achieved with the small number of iterations (Figure 4) is comparable to those reported in the paper by Rueda and Diaz-Uriarte. Hence we believe in the cases where all five CNA segments share the same copy number, the suboptimal performance was mainly caused by the lack of the reference-setting "normal.reference" parameter and the tuning "normal.ref.percentile" parameter. Nonetheless, we agree that for the complex CNA cases, the reduced iterations will result in suboptimal performance.

The authors criticized us for conducting simulations using existing methods "not in the original formulation of their authors". We would like to point out that in Supplement Table 1 of our paper [1], the comparison of all the tested methods were performed with the default settings, except the number of permutations in DNAcopy and number of iterations in RJaCGH. In the results reported in Figure 3 of  [1], modifications of the parameters were made in an effort to adapt them better to the SNP array data and improve their performance. Certainly this was done from a user's perspective and we could not guarantee optimality for every package tested. All the parameters we used were listed in Table 1 of our paper [1]. These manipulations were necessitated by the fact that copy number data generated by the Affymetrix arrays have different characteristics than traditional array-CGH data. It would be beneficial if authors of the packages could provide users guidance as to how to optimize their packages for newer arrays. In general, we are happy that changes are now implemented in the new version of RJaCGH as targeted improvements. And it is encouraging to see the boosted performance of the method on SNP array-based copy number data.
